# Preventable neonatal mortality in the state of São Paulo: a spatial approach

**DOI:** 10.1590/1984-0462/2025/43/2024294

**Published:** 2025-11-14

**Authors:** Julia Ferreira Gomes Pereira, Luiz Fernando Costa Nascimento

**Affiliations:** a Universidade de Taubaté, Taubaté, SP, Brazil.; b Universidade Estadual Paulista, Guaratinguetá, SP, Brazil.

**Keywords:** Neonatal mortality, Spatial analysis, Antenatal care, Apgar score, Perinatal care, Mortalidade neonatal, Análise espacial, Assistência pré-natal, Índice de Apgar, Assistência perinatal

## Abstract

**Objective:**

Identify spatial patterns for preventable neonatal deaths in municipalities in the state of São Paulo between the years of 2015 and 2019, looking for possible correlations with socioeconomic and demographic indices.

**Methods:**

This is an ecological study, with data obtained from the Department of Informatics of the Unified Health System (DATASUS), from the of Mortality Information System (SIM) and Live Birth Information System (SINASC), regarding preventable deaths due to adequate care for women during pregnancy, childbirth and for the newborns, were analysed. Proportions per thousand live births were built. The independent variables used were the proportion of adolescent mothers; insufficient number of antenatal consultations (0–6 consultations); low birthweight (500–2499 g); low Apgar score in the 1^st^ and 5^th^ minute of life (0–7), all of which were used to calculate the Univariate Moran Index (I_MU_). The Social Vulnerability Index (SVI) and the Municipal Human Development Index (MHDI) were used to calculate the Bivariate Moran Index (I_M_). The Univariate and Bivariate Moran’s indexes were calculated, thematic maps and box maps were constructed, and a significance level of α < 5% was adopted.

**Results:**

There were four thematic maps, and three box maps created to analyse the dependent variables, mentioned above. SVI and MHDI, inadequate antenatal care and reduced Apgar score at the 1^st^ and 5^th^ minute showed I_M_ significant with preventable deaths in the neonatal population. A concentration of preventable deaths was identified in the southern region of the state.

**Conclusions:**

The data presented can support municipal managers, demonstrating the need for investment in maternal and child health.

## RESUMO

**Objetivo:**

Identificar padrões espaciais para óbitos neonatais evitáveis em municípios do estado de São Paulo entre os anos de 2015 e 2019, buscando possíveis correlações com índices socioeconômicos e demográficos.

**Métodos:**

Trata-se de um estudo ecológico, com dados obtidos do Departamento de Informática do Sistema Único de Saúde (DATASUS), dos sistemas de informação Sistema de Informações sobre Mortalidade (SIM) e Sistema de Informação sobre Nascidos Vivos (SINASC), sobre óbitos evitáveis por adequada atenção à mulher na gestação, parto e ao recém;-nascido. Foram analisadas e construídas proporções por mil nascidos vivos. As variáveis independentes utilizadas foram a proporção de mães adolescentes; número insuficiente de consultas de pré-natal (0–6 consultas); baixo peso ao nascer (500–2499 g); baixo índice de Apgar no 1º e 5º minuto de vida (0–7), todas utilizadas para o cálculo do Índice de Moran Univariado (IMU). Foram utilizados o Índice de Vulnerabilidade Social (IVS) e o Índice de Desenvolvimento Humano Municipal (IDHM) para o cálculo do Índice de Moran Bivariado (IMB). Foram calculados os Índices de Moran Univariado e Bivariado, foram construídos mapas temáticos e box maps, e foi adotado o nível de significância de α<5%.

**Resultados:**

Foram criados quatro mapas temáticos e três box maps para analisar as variáveis dependentes supracitadas. IVS e IDHM, pré-natal inadequado e Apgar reduzido no 1º e 5º minuto apresentaram IMB significativo com óbitos evitáveis na população neonatal. Foi identificada uma concentração de óbitos evitáveis na Região Sul do estado.

**Conclusões:**

Os dados apresentados podem subsidiar os gestores municipais, demonstrando a necessidade de investimentos na saúde materno-infantil.

## INTRODUCTION

 Since 1990, deaths related to the perinatal period, mainly from preventable causes such as prematurity, have remained as the leading cause of infant mortality in Brazil. Currently 81% of infant deaths occur in the 1^st^ month of life, especially in the early neonatal period (zero to six days of life).^1^ In the state of São Paulo, a study observed that, between 2008 and 2017, preventable neonatal deaths were responsible for most deaths for children up to six days of life, accounting for 72 to 75% of the total of these deaths.^2^

 Preventable deaths are those that are partially or totally preventable by health service actions that are accessible to that population in a given place and time.^3,4^ Since 1976, when the term "preventable death" was coined by Rutstein et al.^5^ monitoring these deaths has been considered of great importance, as they are seen as sentinel events and sensitive indicators of the quality of health services provided in that region.^6,7^ In Brazil, Malta and Duarte, together with the Ministry of Health, revised the issue of preventable deaths in accordance with the Brazilian reality, publishing the "List of preventable causes of deaths due to interventions of the Brazilian Health System". From this list, the preventable causes that can be reduced by adequate care for women during pregnancy, childbirth, the fetus and the newborn were defined.^3,4,8^

 Spatial analysis is a tool that can facilitate the mapping of health events and risk factors across space and time. This approach reveals significant geographic disparities in mortality and healthcare access by identifying vulnerable areas, thereby informing strategic planning and resource allocation. This allows the identification of risk conditions originating from adverse socioeconomical background, and the correlation of these conditions to spatial units and therefore the mortality indexes.^9^ Mohamoud et al.^10^ described the importance of the correlation of neonatal deaths and some variables that are more associated in this period, such as healthcare, nutrition, housing conditions and social services, which helps to reveal in a more precisely way which areas are more vulnerable and what actions could be taken.^3,11,12^

 The aim of the study is to identify spatial patterns for preventable neonatal deaths in municipalities in the state of São Paulo and possible correlations with socioeconomic and demographic indices. 

## METHOD

 This is an ecological and exploratory study that uses spatial analysis tools to estimate and analyze preventable deaths in the 644 of the 645 municipalities from the State of São Paulo between January 2015 and Dezember 2019, which precedes the Covid-19 pandemic in 2020. Ilhabela was removed from the analysis due to an error in the calculation of Moran’s index caused by the lack of contiguity with other cities. The state of São Paulo has a population of 44.4 million inhabitants and a Gross Domestic Product (GDP) of R$2.3 trillion, both the highest national indices. Among the Brazilian states, São Paulo is ranked second according to the Municipal Human Development Index (MHDI), with 0.783, and has a *per capita* income of R$ 1,084.46, also the second highest in Brazil.^13^

 Data relating preventable deaths were obtained from the Mortality Information System (SIM), of the Department of Informatics of the SUS (DATASUS). The data were selected from the "deaths due to preventable causes — 0 to 4 years" database, organized by municipality on the y-axis and by subtype of preventable cause on the x-axis of the table, as well as arranging deaths according to maternal residence. For the analyses, the dependent variables "preventable by adequate care for women during pregnancy (1.2.1)", ‘preventable by adequate care for women during childbirth (1.2.2)’ and ‘preventable by adequate care for the fetus and newborn (1.2.3)’ (for those with zero to 27 days of life and subdivided into early deaths [0–6 days] and late deaths [7–27 days]) were selected and calculated per thousand live births (LB). The data of all the recorded births was obtained from the Live Birth Information System (SINASC) and organized according to maternal residence. The following independent variables were selected for the analysis: maternal age, considering those that occurred in the 10–19 age group; insufficient number of antenatal consultations (0–6 consultations); low birthweight (500–2499 g); low Apgar score at 1st and 5^th^ minute of life (0–7) and their respective percentages. The socio-economic variables selected for the study were the Social Vulnerability Index (SVI) and the MHDI from 2010, provided by the Institute for Applied Economic Research (IPEA). 

 For the spatial analysis, the Terraview 4.2.2 software was used, provided free of charge by National Institute for Space Research (INPE), in which the Moran Global Index (I_M_) was calculated, thematic maps were created for each variable studied and box maps were created from the Local Moran’s Index (LISA). Moran’s Coefficient indicates spatial autocorrelation and is contained in a range between [+1 and -1]. Values close to zero indicate that there is no significant spatial autocorrelation (mosaic map). Values closer to +1 or -1 indicate positive autocorrelation (formation of clusters) or negative autocorrelation (formation of islands), respectively. The box maps are constructed based on the normalized values, in which the attribute was classified according to its position in relation to the quadrants of the Moran Scatter Diagram and given a specific classification where the values of the deviation of the attributes in relation to the average (Z) are associated with the X axis, and the value of the average of its neighbors (WZ) with the Y axis. The High-High classification is given to cities with high rates (*high*) surrounded by cities with high rates (*high*), with a higher control priority and Low-Low for cities with low rates (*low*) surrounded by cities with low rates (*low*), with a low priority. The GeoDa v. 18 program was also used to calculate the Bivariate Moran’s Index (I_M_), with the dependent variable being the proportion or rate of deaths according to the group to which it belongs and the independent variables. The significance level adopted was α=5%. As this is an ecological study using secondary data available to the general public, submission to the Ethics Committee was waived. 

## RESULTS

 From 2015 to 2019, 3,036,603 births were identified in the state of São Paulo; according to the SIM, there were 23,153 neonatal deaths in the state of São Paulo, of which 16,657 (71.94%) were due to preventable causes. Considering these causes, 8,408 deaths (2.76/1000 LB) would have been prevented by adequate care for women during pregnancy, 2,916 (0.96/1000 LB) by adequate care for women during childbirth and 5,333 (1.75/1000 LB) by adequate care for the fetus and newborn. Of the preventable deaths, 12,045 (3.96/1000 LB) occurred in the early age group, zero to six days of life, and 4,612 (1.51/1000 LB) occurred in the late age group, seven to 27 days of life. 

 The data for the independent variables can be seen in Table 1. The proportion of ignored data did not reach 2% of any of the values analyzed. 

**Table 1:** Independent variables and their respective percentages. State of São Paulo, 2015–2019.

Independent variables	n (%)
Adolescent mothers	369,674 (12.17)
Inadequate number of antenatal consultations	628,189 (20.68)
Low birth weight	275,453 (9.07)
Low Apgar score in the first minute	339,653 (11.18)
Low Apgar score in the fifth minute	57,983 (1.90)

 The results showed a concentration of neonatal deaths preventable by adequate care for women during pregnancy (1.2.1) (50.47%), followed by adequate care for the fetus and newborn (1.2.3) (32.02%), and lastly, prevented by adequate care for women during pregnancy (1.2.2) (17.51%). 

 Calculating the Univariate Moran’s Index (I_MU_) on the variables analyzed (Table 2), "preventable deaths by adequate care for the fetus and newborn (1.2.3) per thousand live births", "% of teenage mothers", "% inadequate antenatal", "% low birth weight", "% low Apgar score in the 1st minute" and "% low Apgar score in the 5^th^ minute" were variables considered statistically significant (p<0,05), meaning that these municipalities have an influence on the results obtained on the neighboring cities. Two other variables, "preventable deaths due to adequate care for women during pregnancy (1.2.1) per thousand live births" and "preventable deaths due to adequate care for women during childbirth (1.2.2) per thousand live births", were not statistically significant. 

**Table 2:** Univariate Moran Index (I_MU_) and p-value of the studied variables. State of São Paulo, 2015–2019.

Variables	I_MU_ (p-value)
Preventable deaths by adequate care for women during pregnancy/1000 LB	0.032 (0.096)
Preventable deaths by adequate care for women during childbirth/1000 LB	0.009 (0.282)
Preventable deaths by adequate care for the fetus and newborn/1000 LB	0.069 (0.007)
% of teenage mothers	0.269 (0.001)
% inadequate antenatal	0.392 (0.001)
% low birth weight	0.162 (0.001)
% low Apgar score in the 1^st^ minute	0.368 (0.001)
% low Apgar score in the 5^th^ minute	0.296 (0.001)

LB: live births.

**Figure 1:**
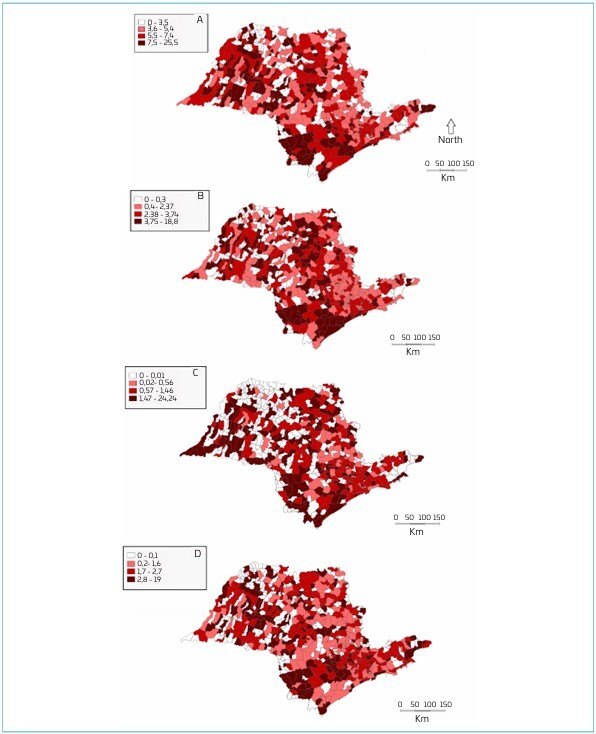
Thematic map of the preventable deaths (A: total; B: by adequate care for women during pregnancy; C: by adequate care for women during childbirth; D: by adequate care for the fetus and newborn) per thousand live births, State of São Paulo, 2015–2019.

 The thematic map of the rate of total preventable deaths per thousand live births (Figure 1A) shows that there is a high concentration of deaths in the southwest of the state, in the Itapeva region, as well as in the northwest of the state in the Rancharia region, with an emphasis in the city of São Pedro do Turvo. 

 Analyzing the variable "preventable deaths due to adequate pregnancy care" (Figure 1B), it is possible to identify a concentration of deaths in the south of the state, again in the Itapeva region and also in the region between Registro and Itanhaém, especially in the city of Birigui. 

 For "preventable deaths due to adequate care for women during childbirth" (Figure 1C), there is a higher incidence in the west of the state, both in the south, in the Itapeva region, and in the north, in the region between Rancharia and Teodoro Sampaio, with an emphasis in the city of Poloni. At last, the map for "preventable deaths due to adequate care for the fetus and newborn" (Figure 1D) is similar to the other causes, with a high incidence of deaths in the south of the state in the Itapeva region and in the north in the Birigui region, with higher rates in the city of Arco-Íris. 

 The Bivariate Moran’s index values for the socioeconomic indices and the percentages of independent variables with their respective dependent variables per thousand live births are shown in Table 3. The socioeconomic variables had statistically significant spatial autocorrelation with the three dependent variables. The independent variable "percentage of teenage mothers" was significantly autocorrelated with the variable "preventable deaths due to adequate care for women during pregnancy", as were the independent variables "percentage of low 1^st^ minute Apgar score" and "percentage of low 5^th^ minute Apgar score". The independent variable "percentage of low birth weight" was significantly autocorrelated with the dependent variable "preventable deaths due to adequate care for women during childbirth", as were the independent variables "percentage of inadequate antenatal care", "percentage of teenage mothers" and "percentage of low 5^th^ minute Apgar score". The variable "preventable deaths due to adequate newborn care" was statistically significant and spatially correlated with "percentage of adolescent mothers" and "percentage of low 5^th^ minute Apgar score". Based on the data obtained from the Local Index of Spatial Association — LISA (Local Moran’s Index), three box maps of the respective causes of preventable deaths were constructed. 

**Table 3:** Bivariate Moran Index (I_M_) and p-value for the independent variables correlating with their respective dependent variables per thousand live births. State of São Paulo, 2015–2019.

Independent variable	Preventable deaths by thousand live births by		
	Adequate care for women during pregnancy (p-value)	Adequate care for women during childbirth (p-value)	Adequate care for the fetus and newborn (p-value)
SVI	0.074 (0.002)	0.053 (0.002)	0.042 (0.018)
MHDI	-0.036 (0.028)	-0.032 (0.036)	-0.045 (0.014)
Low birth weight (%)	-0.009 (0.328)	-0.036 (0.035)	-0.011 (0.245)
Inadequate prenatal care(%)	-0.010 (0.322)	0.029 (0.048)	0.004 (0.460)
Teenage mothers (%)	0.056 (0.002)	0.066 (0.002)	0.049 (0.010)
Low Apgar score in the 1^st^ minute (%)	0.053 (0.006)	0.066 (0.002)	0.049 (0.010)
Low Apgar score in the 1^st^ minute (%)	0.053 (0.06)	0.010 (0.282)	0.023 (0.115)

SVI: Social Vulnerability Index; MHDI: Municipal Human Development Index.

**Figure 2:**
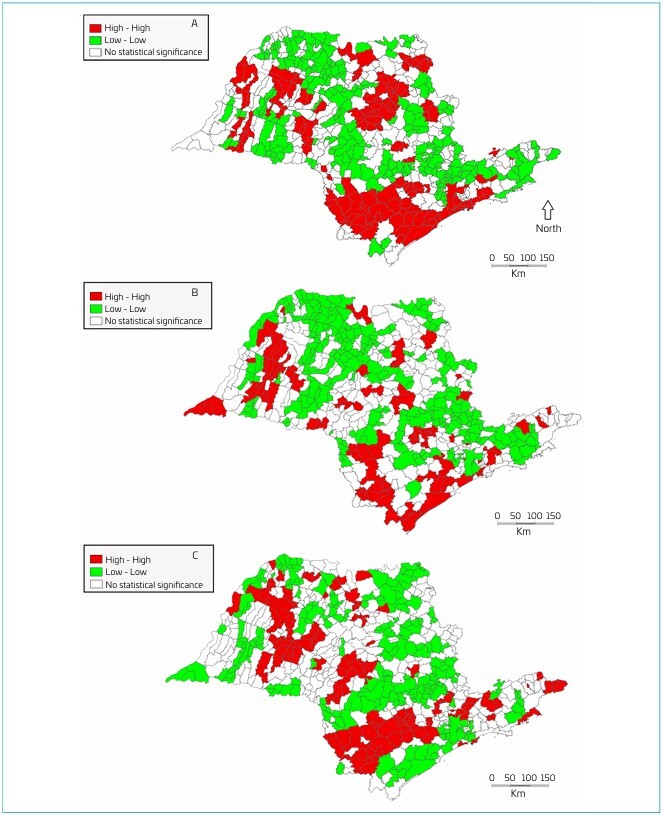
Box Map of the preventable deaths (A: by adequate care for women during pregnancy; B: by adequate care for women during childbirth; C: by adequate care for the fetus and newborn) per thousand live births, State of São Paulo, 2015–2019.

 Looking at the map of "preventable deaths due to adequate care for women during pregnancy" per thousand live births (Figure 2A), it is possible to see a large concentration of cities considered to be high risk in the south of the state, between the city of São Paulo and Itapeva, and in the north of the state in the region of Ribeirão Preto, Araraquara and Marília, totaling 144 classified as "High-High". On the other hand, 240 cities are classified as low risk, in the regions of Jundiai, Guaratinguetá, Bauru and São José do Rio Preto. 

 In the box map of "preventable deaths due to adequate care for women during childbirth" per thousand live births (Figure 2B), there are 102 cities considered to be high priority for municipal managers. There is a focus on the south coast of São Paulo, such as Iguape and Itanhaém, also in the southwest in the region of Itapeva and further north in the region of Presidente Prudente. Two hundred and fifty-nine cities were classified as "Low-Low", including the city of São Paulo, the Paraíba Valley, such as São José dos Campos, the Campinas region, the Itapetininga region, the Marília region and the São José do Rio Preto region. 

 The box map of preventable deaths due to adequate care for the fetus and newborn per thousand live births (Figure 2C) shows that 124 municipalities are classified as "High-High", including the Itapetininga region, the Bauru region and the Araçatuba region. On the other hand, 217 cities were classified as low priority, including the city of São Paulo, the south coast of São Paulo, the Piracicaba region and the São Carlos region. 

## DISCUSSION

 The results showed a concentration of neonatal deaths preventable by adequate care for women during pregnancy, followed by adequate care for the fetus and newborn, and lastly, prevented by adequate care for women during pregnancy. The spatial analysis showed a concentration of preventable deaths in the southern region of the state, especially in the Itapeva region. The socioeconomic variables, percentage of inadequate antenatal care, and low Apgar score at the 1^st^ and 5^th^ minute showed significant autocorrelations with preventable deaths in the neonatal population. The box maps indicated the high-priority areas concentrated mainly in the south and southwest of the state. 

 Although this study did not find statistically significant autocorrelations between the number of antenatal care visits and preventable deaths due to adequate care for women during pregnancy, some of the main causes of preventable deaths, such as prematurity, maternal factors, infections, asphyxia and congenital anomalies, can be detected early with adequate and integral care, which can significantly reduce infant morbidity and mortality. Tomasi et al. stated that a high-quality antenatal care can reduce maternal and child morbidity and mortality, since it allows appropriate guidance and referrals at each stage of pregnancy.^14^ Furthermore, the Brazilian Ministry of Health states that adequate antenatal care and care during childbirth are key factors to reduce neonatal mortality and the prevalence of perinatal conditions.^15^

 Although no spatial autocorrelation was found with preventable deaths due to adequate care for women during pregnancy, preventable deaths due to adequate care for women during childbirth correlated statistically significantly with the insufficient number of antenatal care visits. This finding is supported by the studies done by Ota and Rosa-Mangeret, who stated that a reduced number of antenatal consultations probably results in an increase in perinatal deaths.^16,17^

 Another important factor correlated with inadequate antenatal care is the newborn with low birthweight. According to Rosa-Mangeret, neonates with low birth weight have an increased risk of mortality, delayed neuropsychomotor development and the occurrence of diseases in adulthood.^17^ In addition, Rodríguez states that is a determining factor in infant mortality in the neonatal period, together with biological, social, and maternal factors, among others.^18^ Although no statistically significant spatial autocorrelation was found between preventable deaths due to adequate care for women during pregnancy and low birth weight, a 2019 study correlated low birth weight with deaths that could be reduced due to better care for pregnant women, in which 56.3% of deaths in newborns with low birth weight were classified as reducible due to this cause.^19^ Moreover, a study carried out in Rio Grande do Sul analyzing preventable infant deaths noted that most deaths occurred in the early neonatal period, with a major influence from complications related to prematurity. The text mentions the possible influence of the excessive medicalization of births and the significant increase in caesarean sections, which in the state studied grew in incidence concomitantly with the increase in premature births, especially in the wealthiest families.^20^ Another article discusses that less than 30% of women prefer child delivery by cesarean section at the beginning of pregnancy, but 90% of births result in cesarean procedures, even though only 10% of cases were medically indicated.^21^ This could explain the results observed, since mothers choosing to have caesarean sections, even without a medical benefit and with the risk of harm to the baby, may influence weight at birth and, consequently, the spatial distribution of this occurrence. 

 Another aspect to consider that may be linked to underweight births is maternal age. In addition to the possible interference with the infant’s weight, teenage pregnancy is an important risk factor for maternal and child health problems, as described by a study published in 2014, in which pregnant teenagers had a 50% increased risk of stillbirths and neonatal death, as well as an increased risk of premature birth, low birth weight and asphyxia, compared to pregnancies of older women.^22^ In this study, the Bivariate Moran Index (I_M_) analysis showed a positive correlation between municipalities with a high percentage of mothers under the age of 19 and preventable deaths due to adequate care for women during pregnancy. Both variables showed higher incidence rates in the south of the state, on the southern coast of São Paulo and in the southwestern part of the state, in the Itapeva region. 

 The socioeconomic variable showed spatial correlations with all the dependent variables studied. Considering the SVI, there was a positive spatial autocorrelation, that is, the greater the social vulnerability of that location, the higher the rates of preventable deaths. The MHDI showed something similar, with a negative spatial correlation, indicating that municipalities with lower indices would have higher rates of preventable deaths. A study carried out in Paraná State in 2020 obtained similar findings as this article, where the perinatal mortality rate exhibited inverse spatial autocorrelations with the socioeconomic variables MHDI, degree of urbanization, and proportion of economically active population over 18.^23^ In addition, Bugelli et al., in their multilevel analysis study, found negative correlations between households with family income above two minimum wages and positive correlations with households with family income below this parameter with the neonatal mortality; thus, it was found that the higher the family income, the lower the neonatal mortality, and the reverse applied to families with lower income, where higher rates were found.^24^ In order to observe the possible impacts of the poor distribution of healthcare and spending cuts on maternal and child health, a study observed that low-cost screening and treatment could have reduced by 35.3% the preventable infant deaths during prenatal care if the mothers had received proper care and treatment for conditions such as hypertension, urinary tract infection, vaginosis, gestational diabetes, or previous diabetes.^25^ Another study observed that, in low and middle-income countries, greater public expenditures on health, expanded coverage of maternity services, and number of skilled health care workers were significantly associated with lower neonatal mortality. However, there is a contradiction between those findings and the association with lower inequality neonatal mortality rates, calculated according to the World Health Organization (WHO) Handbook on health inequality monitoring.^26^ This means that areas with lower socio-economic indices have inadequate access to quality health care. 

 After analyzing preventable deaths due to adequate care at birth (1.2.2) and for the newborn (1.2.3), a positive correlation was found with the variables low Apgar score at the 1^st^ and 5^th^ minute. Although the Apgar score itself does not diagnose perinatal asphyxia, low scores in the 5^th^ minute are an important predictor of early neonatal death, as discussed in the article by Rosa-Mangeret.^17^ According to Tavares, a low Apgar score is an important correlating factor with the increase of mortality in neonates without congenital anomalies.^27^ Thus, the regions of Marília, Itapetininga, Bauru and Barretos need an intervention to identify possible causes and reduce the incidence of preventable deaths through adequate care for childbirth (1.2.2) and the newborn (1.2.3). 

 From the findings of the Moran’s Index, in the univariate analysis of deaths, despite not having an identical distribution, the three variables pointed to the southwest of the state, more precisely the Itapeva region. As for the results obtained with the box map, the worst indexes can also be seen further to the south of the state in the administrative region of Itapeva. This region encompasses the Ribeira Valley, one of the most vulnerable regions in the states of São Paulo and Paraná, with an average HDI of 0.75, lower than the state averages,^28^ and with part of the population living under highly vulnerable conditions — considering that 25.91% live in rural areas and 7.65% live under extreme poverty conditions.^29^ These findings correlate with the statistical significance found in the Bivariate Moran test when analyzing the association between socioeconomic indices and preventable neonatal deaths. 

 This study had limitations related to the use of secondary data, such as underreporting, diagnostic errors, or incorrect addresses. Further studies on this subject are needed to better explore factors not mentioned in this article, such as maternal conditions, gestational hypertension, diabetes mellitus, urinary infections, as well as postpartum complications not related to preventable deaths. 

 In conclusion, socioeconomic variables SVI and MHDI, percentage of inadequate antenatal care, and percentage of low Apgar score in the 1^st^ and 5^th^ minute showed significant autocorrelations with preventable deaths in the neonatal population. The data presented in this study can serve as a basis for municipal managers, especially in the southern region of the state, to investigate these causes and possibly invest more financial resources in maternal and child health. 

## Data Availability

The database that originated the article is available with the corresponding author.
